# Childhood Obesity: Does it Have Any Effect on Young Arteries?

**DOI:** 10.3389/fped.2020.00389

**Published:** 2020-07-16

**Authors:** Andrea Emese Jakab, Erzsébet Valéria Hidvégi, Miklós Illyés, Attila Cziráki, Tibor Kalmár, Zoltán Maróti, Csaba Bereczki

**Affiliations:** ^1^Department of Pediatrics, Albert Szent-Györgyi Health Center, University of Szeged, Szeged, Hungary; ^2^Heart Institute, UP Clinical Centre, University of Pécs, Pécs, Hungary

**Keywords:** children and adolescents, overweight, obesity, arterial stiffness, pulse wave velocity, augmentation index, central blood pressure

## Abstract

Prevalence of overweight (OW) and obesity (O) in children and adolescents has been increased in the past three decades. Increased arterial stiffness measuring by aortic pulse wave velocity (PWV_ao_) might be detected in OW/O children and adolescents. The aim of our study was to compare the arterial function parameters (AFPs), such as PWV_ao_; aortic augmentation index (Aix_ao_); aortic systolic blood pressure (SBP_ao_) and brachial systolic blood pressure (SBP_brach_) measured simultaneously in O/OW patients and healthy subjects. In our study 6,816 subjects (3,668 boys) aged 3–18 years were recruited and categorized by their body mass index (BMI) into normal weight (N), OW and O groups regarding their age and sex. AFPs were measured by a non-invasive, occlusive-oscillometric device. 19.9% (*n* = 1,356) of the population were OW/O, 911 (516 boys) were OW and 445 (272 boys) were O. After accounting for the effect of covariates, PWV_ao_ did not differ significantly between N (5.9 ± 0.8 m/s) and OW patients (5.9 ± 0.8 m/s); and N (6.0 ± 0.7 m/s) and O patients (6.0 ± 0.8 m/s). Aix_ao_ was significantly lower in OW (9.3 ± 7.4% vs. 7.6 ± 7.0%, *p* < 0.00001) and in O patients (9.7 ± 8.1% vs. 6.6 ± 7.2%, *p* < 0.00001) compared to controls. No significant difference was found regarding SBP_ao_ values between controls and OW and O groups (*N* = 110.7 ± 12.4 mmHg vs. OW = 110.3 ± 11.9 mmHg; *N* = 115.6 ± 14.0 mmHg vs. O = 114.3 ± 12.8 mmHg). According to our results we may conclude that the unchanged PWV_ao_ in O/OW subjects might be due to the compensatory decrease in Aix_ao_, referring to enhanced vasodilatory status in the studied population.

## Introduction

Prevalence of overweight (OW) and obesity (O) in children and adolescents has increased in both genders in the past three decades (1). Obesity is associated with significantly decreased life expectancy (2), most cardiovascular (CV) deaths are attributed to OW and O (3). Obese children are prone to develop early CV morbidity and are at increased risk for CV mortality during their adult life (4).

Non-invasive measuring of arterial function parameters (AFPs)—such as aortic pulse wave velocity (PWV_ao_), aortic augmentation index (Aix_ao_) and aortic systolic blood pressure (SBP_ao_)—is a widely accepted and recommended in CV risk stratification in adults (5), as these parameters proved to be strong, independent predictors for CV morbidity and mortality (6–8).

Numerous works (cc. 400) (9) were published concerning the relationship between childhood OW/O and arterial stiffness. Evaluating these publications several studies were using local stiffness, measuring regional PWV [e.g., carotid (10–12), radial (13, 14), and tibial (15)]. However, the local and some regional PWV measurements provide information about only the stiffness at the actual point (carotid) and/or a segment (radial, brachial tibial) of the measurement. Furthermore, only carotid-femoral (cfPWV) or PWV_ao_ proved to be a powerful marker to predict the CV morbidity and mortality in adult humans (8, 16). Therefore, papers where not PWV_ao_ or cfPWV were measured, we considered irrelevant from our aspect. Furthermore, PWV_ao_ rises with age, especially after the beginning of puberty in both sexes (17, 18). In this age range, the slope of the increase in the PWV_ao_ is noticeable, thus merely 2 months difference in age among adolescents shows a marked, significant increase in PWV_ao_ (18). Consequently, if we would like to compare PWV_ao_ in OW/O subjects with their N (normal weight) pupils, the identical age should be crucial. Obesity in children and adolescents is characterized by chronic sympathetic overdrive. Sympathetic overactivity occurs in obese patients, as evidence of increased heart rate, blood pressure and cardiac output (19). Moreover, AFPs are strongly influenced by the actual brachial systolic blood pressure (SBP_brach_) (20), thus SBP_brach_ should be the same in the investigated OW/O patients and controls (normal weight, *N*). After a careful review of the available scientific literature, where the sex ratio of the studied population was given, the mean age and the SBP_brach_ of OW/O patients and control groups was identical, only the most relevant PWV_ao_ or cfPWV measurement was performed, no paper fulfilled these rigorous criteria.

Therefore, the aim of our study was to compare the PWV_ao_, Aix_ao_, SBP_ao_, and SBP_brach_ measured simultaneously in OW/O patients and healthy subjects in a population of the wide age range (3–18 years), where the patients and control groups were accurately matched by age, sex and SBP_brach_ to eliminate their effects on AFPs. We intended to apply an easy-to-use, user-independent occlusive-oscillometric device, which enabled us to expand AFP measurements even into a very young (from 3 years) and large population.

## Materials and Methods

### Subjects

6,816 subjects (3,668 boys) aged 3–18 years were recruited from elementary, primary, and high schools in Szolnok town (Hungary) between 2012 and 2016. All the subjects were Caucasian without any chronic diseases, and they were not on any regular medications. Informed consent for the measurements was asked for from the parents of the subjects. The protocol was reviewed and approved by the local Institutional Ethics Committee of the University of Pécs, Pécs, Hungary.

### Methods

Anthropometric measurements (body weight and height) of each subject were carried out according to the WHO recommendation (Kern MGB 150K 100 type electronic scale, MSF 200 type steel strip stadiometer, Kern & SOHN GmbH, Germany) (21). Subjects were categorized by their body mass index (BMI) into N (BMI <90 percentile), OW (90 percentile ≤ BMI <98 percentile), and O (BMI ≥ 98 percentile) groups regarding their age and sex as well (22).

A non-invasive, occlusive-oscillometric device (Arteriograph, TensioMed Ltd, Budapest, Hungary) was used to the AFPs measurements, for which the operating procedure, in practice, did not differ from a standard digital blood pressure measurement. Clinical use of the Arteriograph is user-independent, painless and takes only 2 min with a one-cuff method, hence it is well tolerated by the youngest age group as well. Non-invasive and invasive validations and the detailed method of the device have been published previously (23, 24). The device showed the best variance and reproducibility according to comparative studies against applanation tonometry and piezoelectric methods (23). Furthermore, Arteriograph formerly was used in a large cohort to determine the PWV_ao_ and Aix_ao_ values (percentiles) in a normal, healthy population aged between 3 and 18 years (18, 25). Since the device is user-independent, interobserver and intraobserver errors of the sternal notch-pubic bone distance measurements are only relevant, which have been published earlier and proved to be well within acceptable limits (18). Measurements were taken after a 10-min rest in a comfortable, supine position at room temperature in kindergartens, elementary, and high schools.

### Statistics

Data are reported as mean and SD for continuous data. For statistical tests “*p*” value < 0.05 was used for significance. Bivariate correlation was used for testing correlations between the measured parameters and to generate the descriptive statistics of the original data. Statistical analysis was performed with the SPSS 23.0.0.0 statistical package (SPSS Inc., Chicago, Illinois, USA) and R (version 3.4.4) MatchIt (version 3.0.2) package.

### Propensity Score Analysis

Our data indicated that the distribution of age and sex were significantly different in the different weight categories. Furthermore, strong correlations were observed between the anthropometric, hemodynamic, and arterial function parameters (Supplementary Table 1). To account for the effect of covariates on AFPs propensity score matching was carried out using the “Nearest Neighbor” method with the grouping variable weight (BMI category of N, OW, and O) and SBP_brach_, age and sex as variables to match on. The resulting groups contained 911 matched individuals in case of control vs. OW and 445 matched individuals in case of control vs. O cases that had an almost identical distribution of SBP_brach_, age and sex. The plot of the mean of each covariate against the estimated propensity score, separately by weight status is shown in Supplemental Digital Content (Supplementary Figure 1).

## Results

Totally 6,816 subjects (boys *n* = 3,668; 53.8%) were involved in this study. Among boys 516 were OW (14.1%), 272 were O (7.4%), whereas in girls 395 (12.5%) were OW and 173 (5.5%) were O. The total prevalence of OW and O was significantly higher in boys (21.5%) than in girls (18%) in girls (*p* < 0.001). Altogether 19.9% (*n* = 1 356) of the population were OW/O and 80.1% (*n* = 5 460) of the participants were N.

Baseline characteristics of the investigated cohorts grouped by age and weight categories are summarized for boys (Table 1) and girls (Table 2). Bodyweight, height, BMI, SBP_brach_, diastolic blood pressure (DBP_brach_), mean arterial pressure (MAP), pulse pressure (PP) elevated with increasing age in both genders in every weight category, while heart rate (HR) decreased.

**Table 1:** Characteristics for boys.

**Age**	**n/n_**sum**_**	**Weight**	**Height**	**BMI**	**SBP_**brach**_**	**DBP_**brach**_**	**MAP**	**PP**	**HR**	**PWV_**ao**_**	**Aix_**ao**_**	**SBP_**ao**_**
**(years)**		**(kg)**	**(cm)**	**(%)**	**(mmHg)**	**(mmHg)**	**(mmHg)**	**(mmHg)**	**(1/min)**	**(m/s)**	**(%)**	**(mmHg)**
**(A) NORMAL WEIGHT** ***n*** **=** **2880**												
3	53/62	15.8 ± 2.3	104.1 ± 6.4	14.6 ± 1.7	103.7 ± 6.7	61.7 ± 7.8	76.5 ± 7.8	45.5 ± 10.3	97.8 ± 16.1	5.4 ± 0.5	19.2 ± 8.5	97.4 ± 8.0
4	74/88	17.7 ± 2.6	109.2 ± 7.3	14.8 ± 1.6	104.7 ± 8.4	61.0 ± 7.2	76.4 ± 8.1	45.7 ± 8.4	92.5 ± 12.8	5.5 ± 0.6	16.1 ± 6.4	96.3 ± 9.0
5	132/153	20.0 ± 2.7	116.0 ± 6.3	14.8 ± 1.5	107.1 ± 7.9	62.8 ± 6.1	78.5 ± 7.6	46.7 ± 9.7	91.5 ± 12.6	5.4 ± 0.6	15.2 ± 5.8	98.6 ± 9.2
6	193/222	22.2 ± 2.9	122.6 ± 5.8	14.7 ± 1.4	107.1 ± 9.6	62.7 ± 6.8	77.8 ± 7.6	45.7 ± 9.0	87.0 ± 12.4	5.4 ± 0.6	13.9 ± 5.9	97.9 ± 10.2
7	147/187	24.6 ± 3.3	127.4 ± 6.2	15.1 ± 1.4	108.6 ± 8.8	63.0 ± 6.7	78.4 ± 7.0	46.6 ± 8.5	82.7 ± 13.0	5.3 ± 0.6	13.3 ± 6.2	98.7 ± 8.9
8	139/180	27.8 ± 4.2	133.5 ± 7.0	15.6 ± 1.5	108.6 ± 8.7	63.0 ± 6.0	78.2 ± 6.2	45.5 ± 6.7	79.6 ± 13.1	5.4 ± 0.6	13.0 ± 6.5	98.2 ± 8.7
9	159/199	31.2 ± 5.3	138.4 ± 7.4	16.2 ± 1.7	110.8 ± 8.6	65.4 ± 6.7	80.3 ± 6.7	45.9 ± 6.2	79.8 ± 13.9	5.4 ± 0.6	11.3 ± 6.0	100.0 ± 8.7
10	122/181	35.2 ± 5.6	144.1 ± 6.7	16.9 ± 1.8	110.6 ± 8.3	64.5 ± 5.4	80.1 ± 5.9	46.5 ± 7.9	76.5 ± 11.8	5.4 ± 0.6	10.4 ± 5.7	99.6 ± 8.0
11	134/195	37.5 ± 6.6	148.1 ± 8.3	17.0 ± 1.9	111.1 ± 8.2	64.8 ± 6.0	80.3 ± 6.4	46.6 ± 5.9	73.9 ± 11.9	5.4 ± 0.6	11.3 ± 6.4	99.9 ± 7.9
12	170/232	43.6 ± 8.6	156.0 ± 9.4	17.8 ± 2.1	115.6 ± 10.3	65.7 ± 6.8	82.5 ± 7.4	49.9 ± 7.8	77.3 ± 14.5	5.6 ± 0.7	8.1 ± 6.3	102.2 ± 8.9
13	245/331	50.0 ± 8.7	163.5 ± 9.5	18.6 ± 1.9	120.2 ± 10.7	66.4 ± 6.7	84.5 ± 7.3	53.9 ± 9.1	76.9 ± 13.8	5.7 ± 0.7	5.8 ± 5.3	104.7 ± 9.0
14	301/410	55.8 ± 9.1	170.4 ± 9.3	19.1 ± 1.9	123.5 ± 11.3	67.6 ± 6.3	86.3 ± 7.2	55.8 ± 8.8	75.4 ± 13.9	6.0 ± 0.7	5.0 ± 5.7	107.2 ± 10.1
15	304/374	59.6 ± 8.5	174.3 ± 7.7	19.6 ± 2.0	125.0 ± 11.8	68.9 ± 7.1	87.7 ± 8.0	56.1 ± 9.2	72.2 ± 13.1	6.0 ± 0.6	4.9 ± 5.4	108.6 ± 10.4
16	288/351	63.0 ± 8.9	176.6 ± 7.3	20.2 ± 2.2	129.0 ± 13.0	70.5 ± 7.3	90.0 ± 8.5	58.5 ± 9.5	73.3 ± 15.0	6.2 ± 0.6	5.2 ± 6.0	111.9 ± 11.0
17	295/356	65.6 ± 8.8	178.3 ± 7.0	20.6 ± 2.2	130.1 ± 12.9	70.3 ± 7.2	90.2 ± 8.1	59.6 ± 10.4	71.4 ± 13.2	6.3 ± 0.7	5.2 ± 5.8	112.5 ± 10.9
18	124/147	68.2 ± 8.2	179.5 ± 6.7	21.1 ± 2.0	131.7 ± 11.5	68.2 ± 7.6	89.4 ± 7.5	63.1 ± 10.5	71.4 ± 13.8	6.4 ± 0.5	5.3 ± 5.1	113.0 ± 9.9
	2880/3668											
**(B) OVERWEIGHT** ***n*** **=** **516**												
3	8/62	18.4 ± 2.1	101.3 ± 6.5	17.9 ± 0.3	105.0 ± 7.4	61.0 ± 5.3	75.9 ± 6.1	44.9 ± 5.8	98.3 ± 10.7	5.4 ± 0.8	14.2 ± 7.0	95.9 ± 7.5
4	12/88	23.1 ± 4.9	111.7 ± 11.5	18.3 ± 0.5	111.0 ± 9.0	64.5 ± 7.1	81.6 ± 10.1	51.3 ± 11.9	89.8 ± 10.9	5.1 ± 0.6	14.5 ± 6.5	104.1 ± 14.5
5	13/153	25.3 ± 3.4	117.4 ± 6.7	18.3 ± 0.7	113.6 ± 8.5	65.8 ± 6.3	82.2 ± 6.7	49.3 ± 8.9	87.9 ± 12.5	5.3 ± 0.6	15.6 ± 7.2	102.7 ± 7.3
6	12/222	29.7 ± 4.0	127.0 ± 8.1	18.3 ± 0.5	112.5 ± 13.6	62.9 ± 6.7	79.4 ± 8.3	49.6 ± 9.8	85.3 ± 10.5	5.2 ± 0.5	11.2 ± 5.1	99.5 ± 10.6
7	26/187	33.0 ± 3.3	131.7 ± 6.4	19.0 ± 0.7	116.5 ± 9.3	68.2 ± 5.1	84.6 ± 6.2	49.1 ± 7.2	87.6 ± 15.5	5.3 ± 0.7	12.1 ± 6.9	105.0 ± 8.7
8	24/180	36.9 ± 4.6	136.3 ± 7.6	19.8 ± 0.9	112.9 ± 7.5	65.9 ± 4.6	81.5 ± 5.2	47.0 ± 5.0	80.2 ± 10.6	5.2 ± 0.6	9.5 ± 5.2	100.6 ± 6.8
9	20/199	41.5 ± 4.3	141.2 ± 6.6	20.8 ± 1.0	117.7 ± 7.8	65.9 ± 4.7	83.2 ± 5.1	51.8 ± 6.6	76.6 ± 9.4	5.4 ± 0.5	11.5 ± 4.7	105.0 ± 7.0
10	29/181	49.4 ± 5.1	148.7 ± 6.5	22.3 ± 1.1	119.0 ± 9.0	68.5 ± 7.4	85.2 ± 6.4	51.3 ± 9.4	82.7 ± 14.0	5.6 ± 0.8	8.3 ± 6.7	105.9 ± 10.1
11	39/195	53.1 ± 7.3	152.7 ± 8.9	22.7 ± 1.4	121.5 ± 10.2	68.5 ± 6.9	86.1 ± 7.4	53.0 ± 7.2	75.6 ± 13.3	5.6 ± 0.8	8.7 ± 6.4	107.4 ± 8.5
12	39/232	60.4 ± 8.0	159.8 ± 8.8	23.6 ± 1.5	122.6 ± 7.9	67.8 ± 6.5	86.4 ± 6.2	55.6 ± 9.3	78.1 ± 13.2	5.7 ± 0.7	5.3 ± 4.3	107.1 ± 7.8
13	48/331	67.6 ± 7.9	166.6 ± 8.1	24.3 ± 1.5	126.5 ± 10.8	66.9 ± 8.8	87.0 ± 9.3	60.3 ± 9.4	75.1 ± 10.3	6.0 ± 0.7	6.6 ± 7.5	110.8 ± 13.1
14	81/410	74.2 ± 8.9	171.3 ± 8.9	25.2 ± 1.3	131.3 ± 12.9	68.9 ± 7.7	89.7 ± 8.2	62.4 ± 11.1	76.0 ± 14.2	6.3 ± 0.7	4.4 ± 6.0	112.6 ± 10.5
15	52/374	77.5 ± 7.7	174.9 ± 7.1	25.3 ± 1.4	134.9 ± 13.5	70.7 ± 6.4	92.1 ± 7.6	64.2 ± 11.7	73.0 ± 11.3	6.1 ± 0.7	5.5 ± 7.7	116.6 ± 12.1
16	46/351	82.5 ± 7.6	178.0 ± 6.9	26.0 ± 1.1	135.0 ± 13.2	71.8 ± 7.9	92.9 ± 9.1	63.5 ± 9.5	70.9 ± 11.6	6.2 ± 0.6	3.9 ± 6.4	116.4 ± 12.7
17	46/356	83.8 ± 8.6	178.3 ± 8.3	26.3 ± 1.1	137.1 ± 13.2	72.2 ± 7.6	94.0 ± 9.5	65.5 ± 10.4	72.5 ± 12.5	6.5 ± 0.6	4.7 ± 6.2	118.3 ± 12.3
18	21/147	85.1 ± 10.6	178.3 ± 8.5	26.7 ± 1.4	143.2 ± 13.3	74.6 ± 8.4	97.6 ± 9.2	68.6 ± 9.6	77.0 ± 16.5	6.8 ± 0.6	5.0 ± 4.1	122.9 ± 11.9
	516/3668											
**(C) OBESE** ***n*** **=** **272**												
3	1/62	21.0 ± NA	104.0 ± NA	19.4 ± NA	111.0 ± NA	65.0 ± NA	80.0 ± NA	46.0 ± NA	110.0 ± NA	5.1 ± NA	9.7 ± NA	99.0 ± NA
4	2/88	27.5 ± 2.1	117.5 ± 3.5	19.9 ± 0.3	119 ± 21.2	56.5 ± 5.0	77.5 ± 3.5	62.5 ± 26.2	84.5 ± 16.3	4.8 ± 0.1	9.6 ± 1.8	102.4 ± 13.4
5	8/153	34.5 ± 4.3	123.6 ± 4.1	22.6 ± 2.4	128.5 ± 16.0	64.1 ± 8.1	86.6 ± 9.8	68.1 ± 18.2	95.0 ± 16.9	6.0 ± 0.7	10.4 ± 4.5	114.9 ± 15.3
6	17/222	34.8 ± 4.5	124.9 ± 4.5	22.2 ± 1.6	116.9 ± 10.4	62.4 ± 7.1	81.0 ± 7.2	55.7 ± 13.1	85.5 ± 13.0	5.4 ± 0.5	9.6 ± 5.0	103.4 ± 9.4
7	14/187	43.8 ± 5.5	135.8 ± 6.3	23.7 ± 1.9	123.6 ± 12.2	67.0 ± 6.5	85.9 ± 7.2	56.6 ± 11.3	83.6 ± 13.6	5.3 ± 0.6	10.0 ± 6.7	109.4 ± 10.5
8	17/180	49.5 ± 8.4	139.6 ± 8.4	25.3 ± 2.5	120.6 ± 7.8	70.0 ± 4.3	87.0 ± 4.5	50.7 ± 7.7	81.2 ± 11.3	5.5 ± 0.7	9.6 ± 5.3	107.3 ± 5.5
9	20/199	55.2 ± 6.6	145.6 ± 6.4	26.0 ± 1.8	124.5 ± 10.4	69.4 ± 4.3	87.9 ± 5.1	55.1 ± 10.0	86.4 ± 11.7	5.8 ± 0.6	6.7 ± 6.9	109.2 ± 8.3
10	30/181	63.2 ± 9.6	149.7 ± 7.2	28.2 ± 3.6	124.3 ± 10.5	68.3 ± 7.5	87.1 ± 8.2	56.3 ± 7.5	81.7 ± 14.1	5.7 ± 0.7	5.0 ± 4.4	108.8 ± 9.3
11	22/195	70.8 ± 8.6	157.1 ± 7.5	28.6 ± 2.5	130.0 ± 10.8	72.3 ± 7.3	91.6 ± 7.1	57.7 ± 10.6	77.3 ± 12.3	5.9 ± 0.6	5.2 ± 4.5	112.9 ± 8.5
12	23/232	80.3 ± 8.9	162.7 ± 5.6	30.3 ± 3.0	130.0 ± 9.5	67.7 ± 6.6	88.5 ± 6.5	62.2 ± 8.9	81.2 ± 14.6	6.0 ± 0.5	3.3 ± 3.3	110.7 ± 7.7
13	38/331	85.3 ± 11.0	167.0 ± 7.2	30.5 ± 2.6	134.1 ± 14.5	68.3 ± 8.8	90.3 ± 9.4	66.0 ± 12.7	82.8 ± 10.6	6.3 ± 0.8	3.4 ± 8.6	114.5 ± 12.2
14	28/410	91.9 ± 16.0	170.6 ± 9.5	31.4 ± 3.0	132.0 ± 14.5	69.9 ± 9.0	90.6 ± 10.1	62.1 ± 10.0	75.7 ± 8.9	6.4 ± 0.6	4.1 ± 7.8	113.8 ± 12.2
15	18/374	97.7 ± 10.6	173.7 ± 5.9	32.3 ± 3.1	146.3 ± 15.1	76.9 ± 9.4	99.9 ± 10.5	69.4 ± 9.8	72.4 ± 13.7	6.6 ± 0.8	4.0 ± 4.4	125.3 ± 13.1
16	17/351	101.9 ± 10.9	176.3 ± 6.4	32.8 ± 3.2	145.8 ± 15.8	78.1 ± 10.5	101.3 ± 13.1	69.5 ± 10.7	76.3 ± 13.9	6.5 ± 0.9	7.2 ± 5.8	128.2 ± 17.1
17	15/356	104.8 ± 10.5	179.3 ± 6.2	32.6 ± 2.5	146.8 ± 16.1	77.8 ± 7.8	100.9 ± 9.3	69.0 ± 13.6	75.3 ± 16.6	6.7 ± 0.8	4.4 ± 5.9	125.7 ± 12.3
18	2/147	89.0 ± 7.1	171.0 ± 5.7	30.4 ± 0.4	153.0 ± 19.8	84.0 ± 4.2	107.0 ± 9.9	69.0 ± 15.6	72.0 ± 4.2	5.8 ± 0.0	8.1 ± 1.9	134.2 ± 16.6
	272/3668											

*Data are reported as mean ± SD. Aix_ao_, aortic augmentation index; BMI, body mass index; DBP_brach_, brachial diastolic blood pressure; HR, heart rate; MAP, mean arterial blood pressure; PP, pulse pressure; PWV_ao_, aortic pulse wave velocity; SBP_ao_, aortic systolic blood pressure; SBP_brach_, brachial systolic blood pressure*.

**Table 2:** Characteristics for girls.

**Age(years)**	**n/n_**sum**_**	**Weight**	**Height**	**BMI**	**SBP_**brach**_**	**DBP_**brach**_**	**MAP**	**PP**	**HR**	**PWV_**ao**_**	**Aix_**ao**_**	**SBP_**ao**_**
		**(kg)**	**(cm)**	**(%)**	**(mmHg)**	**(mmHg)**	**(mmHg)**	**(mmHg)**	**(1/min)**	**(m/s)**	**(%)**	**(mmHg)**
**(A) NORMAL WEIGHT** ***n*** **=** **2580**												
3	41/49	15.5 ± 2.5	102.0 ± 6.4	14.8 ± 1.2	103.4 ± 8.8	59.8 ± 9.7	74.8 ± 8.0	47.2 ± 13.2	101.8 ± 13.9	5.6 ± 0.7	21.8 ± 8.2	97.7 ± 10.0
4	66/87	17.1 ± 2.5	108.7 ± 6.5	14.5 ± 1.7	106.1 ± 7.7	61.2 ± 6.2	76.9 ± 6.6	46.5 ± 7.9	93.5 ± 11.7	5.4 ± 0.6	18.7 ± 6.2	99.5 ± 9.5
5	82/99	18.5 ± 3.0	114.3 ± 7.3	14.2 ± 1.6	107.7 ± 11.4	62.6 ± 8.0	78.2 ± 9.2	47.0 ± 9.6	94.4 ± 11.1	5.5 ± 0.7	16.3 ± 6.8	99.6 ± 12.5
6	123/148	21.7 ± 3.4	122.4 ± 6.9	14.5 ± 1.5	109.3 ± 11.1	63.5 ± 7.3	79.2 ± 8.7	46.8 ± 8.3	90.1 ± 14.2	5.4 ± 0.7	15.3 ± 6.6	100.1 ± 11.5
7	116/142	23.9 ± 4.9	127.6 ± 7.9	14.6 ± 1.9	110.1 ± 10.8	64.5 ± 7.4	80.2 ± 8.9	46.8 ± 9.0	87.0 ± 13.0	5.5 ± 0.6	14.5 ± 7.3	101.1 ± 12.1
8	94/122	27.4 ± 4.8	133.2 ± 7.4	15.3 ± 1.7	111.1 ± 10.1	64.9 ± 6.2	80.2 ± 7.0	46.1 ± 7.0	85.0 ± 13.5	5.4 ± 0.6	13.1 ± 5.9	100.5 ± 9.2
9	123/157	29.7 ± 5.1	137.6 ± 7.4	15.6 ± 1.7	111.9 ± 10.1	65.2 ± 6.4	80.6 ± 7.1	47.1 ± 8.2	85.2 ± 13.0	5.5 ± 0.7	11.1 ± 5.1	100.5 ± 9.2
10	111/151	34.2 ± 5.8	143.9 ± 7.0	16.4 ± 1.9	113.2 ± 9.9	65.4 ± 5.9	81.4 ± 6.7	47.7 ± 7.0	83.2 ± 12.7	5.6 ± 0.7	10.7 ± 6.3	101.5 ± 8.6
11	104/137	38.2 ± 7.3	149.1 ± 9.4	17.0 ± 2.0	113.7 ± 9.0	66.4 ± 5.9	82.1 ± 6.4	47.4 ± 6.7	81.6 ± 13.7	5.5 ± 0.7	9.7 ± 5.7	101.5 ± 8.1
12	162/218	43.3 ± 7.6	155.7 ± 7.8	17.7 ± 2.0	117.7 ± 12.2	66.9 ± 6.9	83.9 ± 8.1	50.9 ± 8.7	82.4 ± 15.5	5.8 ± 0.6	8.9 ± 6.5	104.1 ± 10.2
13	257/317	49.1 ± 6.7	161.4 ± 6.9	18.8 ± 2.0	119.4 ± 11.7	67.0 ± 6.5	84.5 ± 7.3	52.5 ± 9.5	80.5 ± 14.8	5.9 ± 0.7	7.5 ± 5.6	104.9 ± 10.1
14	301/356	52.0 ± 7.4	164.0 ± 6.6	19.3 ± 2.2	119.9 ± 11.2	68.2 ± 6.5	85.5 ± 7.4	51.7 ± 8.3	78.1 ± 14.4	5.9 ± 0.6	7.4 ± 5.3	105.6 ± 9.7
15	316/374	54.1 ± 7.5	164.8 ± 6.7	19.9 ± 2.2	119.0 ± 10.6	68.8 ± 6.3	85.5 ± 7.3	50.2 ± 7.5	77.4 ± 13.8	5.9 ± 0.6	7.8 ± 5.4	105.3 ± 9.1
16	281/335	54.7 ± 7.2	164.9 ± 6.5	20.1 ± 2.3	120.1 ± 10.5	70.2 ± 6.1	86.9 ± 7.1	50.0 ± 7.7	77.1 ± 13.7	6.1 ± 0.6	8.3 ± 5.6	106.7 ± 9.5
17	271/311	55.4 ± 7.4	166.1 ± 6.4	20.1 ± 2.2	121.2 ± 11.7	69.4 ± 6.7	86.7 ± 7.6	51.8 ± 8.8	76.9 ± 12.1	6.2 ± 0.6	8.6 ± 5.8	107.2 ± 10.2
18	132/145	56.7 ± 6.5	166.0 ± 6.7	20.6 ± 2.0	125.5 ± 13.8	70.9 ± 7.6	89.1 ± 9.1	54.6 ± 9.5	79.6 ± 13.1	6.4 ± 0.5	8.1 ± 5.4	110.6 ± 11.9
	2580/3148											
**(B) OVERWEIGHT** ***n*** **=** **395**												
3	6/49	18.7 ± 3.2	100.5 ± 7.9	18.4 ± 0.6	112.3 ± 6.7	62.3 ± 3.7	79.5 ± 4.3	51.7 ± 11.1	104.7 ± 8.8	5.2 ± 0.6	18.9 ± 7.3	105.0 ± 10.2
4	11/87	20.9 ± 3.6	107.8 ± 8.8	17.9 ± 0.4	109.6 ± 8.2	62.3 ± 6.5	78.2 ± 6.2	47.7 ± 7.6	96.6 ± 17.4	5.2 ± 0.6	14.8 ± 7.2	99.5 ± 5.8
5	11/99	27.4 ± 3.6	122.4 ± 8.5	18.2 ± 0.7	118.2 ± 9.8	69.0 ± 11.9	86.6 ± 12.7	52.6 ± 8.0	94.7 ± 18.5	5.3 ± 0.7	10.9 ± 7.6	107.9 ± 15.4
6	18/148	29.8 ± 4.2	126.4 ± 8.2	18.6 ± 0.8	112.9 ± 12.1	65.1 ± 8.5	81.2 ± 9.3	48.4 ± 7.2	85.2 ± 13.2	5.3 ± 0.5	14.1 ± 8.3	102.3 ± 11.0
7	17/142	32.2 ± 2.6	131.1 ± 4.6	18.7 ± 0.5	114.9 ± 8.7	64.0 ± 5.6	81.1 ± 5.2	50.9 ± 9.2	85.6 ± 8.9	5.2 ± 0.7	10.8 ± 7.8	102.5 ± 9.4
8	15/122	35.9 ± 5.0	134.3 ± 6.7	19.8 ± 1.1	114.9 ± 10.1	66.6 ± 4.8	82.6 ± 5.9	48.3 ± 8.0	79.2 ± 11.9	5.2 ± 0.6	12.4 ± 6.6	103.7 ± 7.6
9	22/157	40.5 ± 5.2	139.3 ± 8.1	20.8 ± 0.8	117.8 ± 10.9	66.0 ± 5.9	83.2 ± 6.9	51.8 ± 8.1	83.5 ± 13.9	5.2 ± 0.8	9.7 ± 7.0	104.4 ± 7.7
10	21/151	47.8 ± 4.5	147.7 ± 6.1	21.9 ± 1.0	121.1 ± 13.6	67.5 ± 8.0	85.7 ± 10.0	54.6 ± 8.5	89.0 ± 16.1	5.5 ± 0.9	5.5 ± 3.7	106.1 ± 12.0
11	22/137	56.1 ± 8.1	155.0 ± 8.9	23.2 ± 1.4	125.2 ± 11.4	66.5 ± 9.6	86.4 ± 10.0	59.7 ± 9.8	86.4 ± 15.4	6.0 ± 0.8	5.2 ± 5.9	108.5 ± 11.8
12	40/218	63.5 ± 7.0	161.0 ± 6.6	24.5 ± 1.7	127.3 ± 11.4	70.5 ± 7.0	89.6 ± 8.1	57.2 ± 8.5	84.4 ± 13.9	5.9 ± 0.7	6.0 ± 4.0	111.4 ± 10.5
13	51/317	64.2 ± 7.6	160.4 ± 9.8	24.9 ± 1.4	127.5 ± 13.4	70.3 ± 7.5	89.4 ± 8.8	57.2 ± 9.6	82.3 ± 13.5	5.9 ± 0.7	7.1 ± 6.2	112.0 ± 11.6
14	43/356	69.0 ± 7.6	163.9 ± 7.2	25.6 ± 1.4	129.0 ± 13.4	71.3 ± 7.6	90.5 ± 8.7	57.7 ± 10.3	79.1 ± 13.4	6.0 ± 0.7	7.4 ± 10.3	113.1 ± 11.8
15	41/374	70.4 ± 6.7	165.0 ± 6.7	25.8 ± 1.3	125.5 ± 11.2	71.6 ± 6.3	89.5 ± 7.2	54.0 ± 9.2	75.3 ± 10.6	6.0 ± 0.7	7.6 ± 4.7	110.7 ± 10.0
16	34/335	73.0 ± 6.7	165.8 ± 6.6	26.5 ± 1.2	131.1 ± 12.6	74.1 ± 7.6	93.0 ± 8.3	57.0 ± 9.8	79.5 ± 13.1	6.1 ± 0.7	6.7 ± 6.4	115.2 ± 11.4
17	33/311	75.5 ± 7.8	166.6 ± 6.9	27.1 ± 1.6	130.9 ± 14.1	72.2 ± 7.6	91.8 ± 9.1	58.8 ± 10.0	74.1 ± 9.6	6.1 ± 0.5	8.2 ± 5.3	115.1 ± 12.3
18	10/145	76.6 ± 7.0	166.3 ± 7.6	27.7 ± 1.6	125.9 ± 10.8	71.5 ± 3.7	89.6 ± 5.7	54.4 ± 8.6	86.5 ± 8.5	6.4 ± 0.6	6.6 ± 2.8	110.4 ± 8.7
	395/3148											
**(C) OBESE** ***n*** **=** **173**												
3	2/49	21.5 ± 0.7	100.5 ± 0.7	21.3 ± 1.0	127 ± 4.2	73.0 ± 2.8	91.0 ± 2.8	54.0 ± 1.4	97.5 ± 12.0	5.9 ± 0.5	13.1 ± 8.9	114.6 ± 0.3
4	10/87	30.4 ± 4.0	113.6 ± 7.1	23.5 ± 2.4	120.7 ± 7.0	63.5 ± 5.4	82.5 ± 5.2	57.2 ± 6.3	99.0 ± 10.3	5.9 ± 0.7	10.2 ± 10.4	106.3 ± 7.0
5	6/99	29.3 ± 5.3	117.0 ± 8.3	21.3 ± 1.9	113.2 ± 10.1	63.2 ± 4.8	79.7 ± 5.7	50.0 ± 8.6	90.0 ± 11.1	5.4 ± 0.6	16.9 ± 11.5	104.5 ± 14.6
6	7/148	30.6 ± 3.2	118.7 ± 4.2	21.6 ± 1.1	112.1 ± 9.4	63.6 ± 5.6	79.6 ± 6.0	48.6 ± 8.5	89.0 ± 7.4	5.4 ± 0.4	12.0 ± 6.2	100.4 ± 7.5
7	9/142	50.2 ± 11.2	128.9 ± 5.5	30.7 ± 8.7	119.0 ± 10.2	66.3 ± 7.2	83.8 ± 6.5	52.7 ± 10.9	88.0 ± 5.9	5.6 ± 0.6	6.5 ± 5.6	104.2 ± 8.8
8	13/122	49.9 ± 4.3	141.7 ± 3.5	24.9 ± 1.8	122.8 ± 8.9	69.2 ± 5.6	87.2 ± 6.1	53.6 ± 6.7	87.5 ± 6.7	5.5 ± 0.5	7.7 ± 4.8	107.7 ± 7.1
9	12/157	54.1 ± 8.4	144.0 ± 8.7	26.0 ± 2.0	126.5 ± 12.4	70.1 ± 7.6	89.0 ± 8.4	56.4 ± 10.0	84.3 ± 10.8	5.7 ± 0.8	9.7 ± 5.4	112.0 ± 10.6
10	19/151	69.5 ± 10.7	154.7 ± 8.5	29.0 ± 3.3	140.2 ± 14.5	73.4 ± 6.1	95.7 ± 8.1	66.8 ± 11.8	94.2 ± 14.5	6.1 ± 0.8	4.2 ± 9.5	121.2 ± 12.6
11	11/137	69.2 ± 8.7	156.8 ± 8.4	28.1 ± 1.8	125.9 ± 10.4	72.3 ± 9.1	90.3 ± 9.3	53.6 ± 6.0	79.3 ± 15.6	5.8 ± 0.8	9.0 ± 7.5	111.8 ± 12.7
12	16/218	80.6 ± 12.2	160.6 ± 7.1	31.4 ± 5.5	132.1 ± 14.2	70.6 ± 7.7	91.1 ± 8.2	61.4 ± 13.2	81.1 ± 15.4	5.9 ± 0.9	9.2 ± 13.0	116.2 ± 12.1
13	9/317	78.6 ± 11.0	162.2 ± 10.4	29.7 ± 1.3	135.7 ± 6.2	71.3 ± 8.2	92.8 ± 6.8	64.3 ± 7.9	86.1 ± 6.7	6.5 ± 0.5	8.4 ± 10.8	118.9 ± 9.1
14	12/356	84.6 ± 11.4	164.1 ± 6.7	31.3 ± 3.0	140.9 ± 19.3	76.3 ± 11.3	97.8 ± 13.3	64.6 ± 11.4	87.4 ± 9.2	6.6 ± 0.7	4.4 ± 5.4	121.8 ± 16.7
15	17/374	90.6 ± 11.6	166.5 ± 7.1	32.6 ± 2.6	137.4 ± 11.9	74.9 ± 5.7	95.7 ± 7.3	62.4 ± 8.5	79.1 ± 11.5	6.4 ± 0.7	6.5 ± 6.6	119.7 ± 8.9
16	20/335	92.5 ± 11.8	165.9 ± 6.0	33.6 ± 3.7	138.9 ± 10.5	78.3 ± 3.8	98.6 ± 5.5	60.7 ± 8.2	81.6 ± 12.1	6.4 ± 0.5	8.6 ± 6.4	122.5 ± 8.9
17	7/311	94.7 ± 10.5	161.7 ± 9.9	36.2 ± 2.1	147.6 ± 14.7	73.3 ± 8.7	98.0 ± 7.7	74.3 ± 17.0	80.1 ± 14.7	6.2 ± 0.8	5.3 ± 3.6	125.7 ± 10.4
18	3/145	102.0 ± 0.0	169.0 ± 0.0	35.7 ± 0.0	133.7 ± 15.0	62.0 ± 9.5	86 ± 11.1	71.7 ± 5.8	57.7 ± 1.2	5.6 ± 0.4	1.3 ± 4.0	110.8 ± 13.5
	173/3148											

*Data are reported as mean ± SD. Aix_ao_, aortic augmentation index; BMI, body mass index; DBP_brach_, brachial diastolic blood pressure; HR, heart rate; MAP, mean arterial blood pressure; PP, pulse pressure; PWV_ao_, aortic pulse wave velocity; SBP_ao_, aortic systolic blood pressure; SBP_brach_, brachial systolic blood pressure*.

Mean HR and SBP_brach_ values showed an increment with increasing BMI categories (Table 3). HR showed a clinically insignificant increased mean value in OW group than in N subjects (79.6 ± 14.1/min vs. 79.5 ± 14.9/min, non-significant). However, the HR was significantly increased in O group compared to N group (82.5 ± 13.5/min vs. 79.5 ± 14.9/min, *p* < 0.0001). The mean HR values were also significantly different in OW and O groups (79.6 ± 14.1/min vs. 82.5 ± 13.5/min, *p* < 0.02). Mean SBP_brach_ was higher in OW, and higher in O patients, than in N subjects (*N* = 117.7 ± 13.1 mmHg, OW = 125.6 n ± 14.0 mmHg, O = 131.2 ± 15.2 mmHg), the differences were significant between every patient groups (*p* < 0.0001).

**Table 3:** Mean heart rate and SBP_brach_ values in N/OW/O patients.

	**N**	**OW**	**O**	N-OW^*^	OW-O^*^	N-O^*^
Number of patients (*n*)	5460	911	445			
Heart rate	79.5 ± 14.9	79.6 ± 14.1	82.5 ± 13.5	NS	*p* = 0.02	*p* < 0.0001
(1/min)				(*p* = 0.98)		
						
SBP_brach_	117.7 ± 13.1	125.6 ± 14.0	131.2 ± 15.2	*p* < 0.0001	*p* < 0.0001	*p* < 0.0001
(mmHg)						

*Data are reported as mean ± SD. HR, heart rate; N, normal weight; O, obese; OW, overweight; SBPbrach, brachial systolic blood pressure*;

* *, Kruskal-Wallis test*.

Characteristics of the propensity score matched groups and clarified AFPs are summarized in Table 4.

**Table 4:** Characteristics of OW and O patients vs. controls and AFP values after propensity score matching.

	**OW**			**O**		
	**Controls**	**Patients**	**Welch**	**Controls**	**Patients**	**Welch**
			***t*-test**			***t*-test**
Number of patients (*n*)	911	911		445	445	
Age (years)	12.7 ± 4.4	12.7 ± 3.8	NS/^*^	11.8 ± 4.6	11.8 ± 3.6	NS/^*^
Sex ratio	520/391	516/395	NS	271/174	272/173	NS
(boys/girls)						
Body height (cm)	155.9 ± 22.9	156.7 ± 20.3	NS *p* = 0.39	155.9 ± 24.6	154.3 ± 18.8	NS *p* = 0.28
Body weight (kg)	46.4 ± 18.0	60.4 ± 19.8	*p* < 0.00001	44.7 ± 19.5	71.5 ± 24.0	*p* < 0.00001
SBP_brach_	124.9 ± 15.1	125.6 ± 14.0	NS	130.8 ± 17.2	131.2 ± 15.2	NS
(mmHg)						
PWV_ao_	5.9 ± 0.8	5.9 ± 0.8	NS	6.0 ± 0.7	6.0 ± 0.8	NS
(m/s)			*p* = 0.68			*p* = 0.66
Aix_ao_	9.3 ± 7.2	7.6 ± 7.0	*p* < 0.00001	10.7 ± 8.0	6.6 ± 7.2	*p* < 0.00001
(%)						
SBP_ao_	110.7 ± 12.4	110.3 ± 11.9	NS	115.6 ± 14.0	114.3 ± 12.8	NS
(mmHg)			*p* = 0.49			*p* = 0.14

*Data are reported as mean ± SD. Aix_ao_, aortic augmentation index; NS, non-significant; O, obese; OW, overweight; PWV_ao_, aortic pulse wave velocity; SBP_ao_, aortic systolic blood pressure; SBP_brach_, brachial systolic blood pressure*;

* *, mean age was significantly different between OW and O group, p < 0.001*.

Mean age was significantly lower in O group (11.8 ± 3.6 ys) compared to OW group (12.7 ± 3.8 ys) (*p* < 0.001). Sex ratio was the same in the investigated groups. The mean body height was almost the same both in OW and O groups compared to controls, the difference was 0.8 cm (NS, *p* = 0.39) and 1.6 cm (NS, *p* = 0.28), respectively. Obviously, the mean body weight was significantly higher in OW and O groups than in controls. Although the O patients were younger, their mean SBP_brach_ was significantly higher (131.2 ± 15.2 mmHg) than was in OW patients (125.6 ± 14.0 mmHg) (*p* < 0.0001).

After the elimination of the alternating effects of age, sex and SBP_brach_, PWV_ao_ values were not significantly different between the propensity score matched N patients (5.9 ± 0.8 m/s) and OW patients (5.9 ± 0.8 m/s) and between *N* patients (6.0 ± 0.7 m/s) and O patients (6.0 ± 0.8 m/s). A significant difference was found between N and OW patients in Aix_ao_, namely, in N patients Aix_ao_ was 9.3 ± 7.4% whereas it was 7.6 ± 7.0% in OW patients (*p* < 0.00001), and Aix_ao_ was higher in N subjects (9.7 ± 8.1%) compared to O patients (6.6 ± 7.2%), which difference proved to be statistically significant (*p* < 0.00001) as well. Regarding SBP_ao_ no significant difference was found between N and OW (110.7 ± 12.4 mmHg vs. 110.3 ± 11.9 mmHg) subjects. In the same way, the difference was not significant between the SBP_ao_ values of propensity score matched N and O subjects (115.6 ± 14.0 mmHg vs. 114.3 ± 12.8 mmHg).

## Discussion

The most important finding of our study is that no difference was found regarding PWV_ao_ between N and OW, N and O patients after accounting for the effect of covariates. Furthermore, we observed a marked, significant decrease of Aix_ao_ in OW, and a markedly more significant decrease in O patients. Finally—similarly to PWV_ao_–no differences were found regarding SBP_ao_ neither in OW, nor in O patients in a properly matched analysis.

### PWV_ao_

With the use of propensity score analysis, no significant difference was found within the different weight categories regarding PWV_ao_. This is in apparent disagreement with previous reports indicated increased PWV_ao_ in O children and adolescents (26, 27). Analyzing the reference values of PWV_ao_ measured in children and adolescents (18), it is apparent that the median percentile curve of PWV_ao_ shows a flat period between the age of three and the beginning of puberty with a steeper increase thereafter in both sexes. Therefore, it is necessary to match the age of the investigated patients and control groups precisely. Only three publications were found in which the mean age of the examined groups were identical (26–28). However, in these studies, SBP_brach_ differed in the different studied patients' groups, i.e., the SBP_brach_ was higher in the OW/O groups compared to the controls. It is well known that the PWV_ao_ is significantly dependent on the SBP_brach_ (20), namely the higher the SBP_brach_, the higher the PWV_ao_. Consequently, in these studies, the elevated PWV_ao_ may originate from increased SBP_brach_. Eliminating the possible modifying factors, age, sex, and SBP_brach_ matched control groups were formed in our study. As a result of this, no significant differences were found regarding PWV_ao_. Charakida et al. published data on PWV in children (over 6,000 subjects) with elevated BMI, however, they measured regional (radial) PWV, not aortic. Unfortunately, predicting the value of regional (brachial, radial, tibial) PWV is not established in cardiovascular morbidity and mortality, so it is considered irrelevant from our aspect (13). Lurbe et al. (29) published AFPs data in overweight and obese children, nevertheless, the difference in age between the investigated groups was more than 1 year, which might lead to misinterpretations in the aspect of AFPs (obese 12 ± 2.2 y vs. overweight 13 ± 2.1 y vs. non-obese 13.4 ± 2.6 y).

### Aix_ao_

Aix_ao_ a measure of central wave reflection, is strongly associated with peripheral arterial resistance (30), which is determined mainly by the tone of small arteries and arterioles. From the cited three studies (26–28) only Mocnik et al. measured Aix, and they found that the Aix was lower in OW/O patients than in controls (28). Unfortunately, it was not published whether Aix_ao_ or Aix_brach_ was measured. Aix_ao_ and Aix_brach_ are correlated very strongly, they are basically identical, only the scaling is different between aortic and brachial Aix (24). Thus, *Mocnik*'s findings support our observed results where we have found the Aix_ao_ to be significantly lower in OW/O patients groups compared to N subjects.

Nevertheless, the enhanced wave reflection in small children is a well-known phenomenon (31), moreover, our working group proved that the increased Aix_ao_ detected in early childhood can only be caused by the shorter body height (aortic length) (25). Therefore, it is possible, that the observed differences regarding Aix_ao_ between N, OW and O groups might stem from the differences in body height. As the mean body height was not significantly different between patients and controls—neither in case of OW, nor in O—we might declare that the observed differences regarding Aix_ao_ are independent of the body height.

Obesity in children and adolescents is characterized by chronic sympathetic overdrive (19). Sympathetic overactivity occurs in obese animals, as evidenced by increased blood pressure, cardiac output, heart rate as well as the activation of the renin-angiotensin-aldosterone system (32). In our study the SBP_brach_, the HR was increased in OW patients and it was significantly higher in O subjects than in controls. This finding could be explained by the previously mentioned chronic sympathetic overdrive; consequently, the increased metabolism will cause a remarkable increase in heart rate, stroke volume and cardiac output. Wilkinson et al. have found an inverse linear relationship between HR and Aix_ao_ (33). Together these findings could explain the observed lower Aix_ao_ in OW/O patients. Presumably, due to the increased cardiac output and the concomitantly increasing SBP_brach_ and SBP_ao_, the lateral tension toward the aortic wall is also increased, the aorta dilates; thus, the aortic wall becomes stiffer and, as its consequence, the PWV_ao_ could be higher. To compensate for the effects of increased cardiac output and SBP_ao_, the vascular regulation system might lower the vascular resistance by dilating the small arterioles; therefore, decreased Aix_ao_ could be detected. This finding might be supported by the observations, which revealed increased nitric oxide (NO) level in relation to fat accumulation (34).

### SBP_ao_

In the overviewed and cited literature SBP_ao_ has not been measured or published. In our study, after the elimination of the effects of sex, age, and SBP_brach_, no significant differences were found regarding SBP_ao_ in OW/O patients and controls. This observation might originate from the accurately matched SBP_brach_ (age and sex also) between patient and control groups, consequently, in this age period, the SBP_ao_ is mainly determined by SBP_brach_, without any detectable morphological or functional deterioration of the aortic wall.

### Study Limitations

Although the use of BMI is well-known and widely accepted technique to identify overweight and obesity, it might be misleading in some cases, because it does not distinguish fat tissue from muscle tissue, therefore individuals with a considerable muscular system may have increased BMI resulting in “artificial” obesity. In our study, age- and sex-based BMI was used to categories patients, still, other techniques such as skinfold measurements, waist-hip ratio, or bioelectrical impedance analysis would be necessary for more proper weight categorization.

To confirm our theory according to which the sympathetic overdrive in OW/O patients resulting in increased cardiac output and elevated SBP_brach_, stroke volume and cardiac output should have been measured by echocardiography in these subjects. In our study, there is a lack of these data, since it was not the aim of this investigation. Detailed history taking on overweight and obesity (onset, duration) and medical history about the closest family members could add relevant information to this study. Finally, there is a lack of lifestyle data (smoking, caffeine intake, eating habits, etc) and detailed laboratory tests (fatty-acid profile, metabolic panel). Future studies of this working group will collect the above-mentioned data in order to elucidate the underlying causes of the observed phenomena and hypothesis.

## Conclusions

Our study comprises anthropometric and AFPs data of a huge cohort of N/OW/O subjects. Interestingly, by comparing OW/O population data with the properly matched identical subset of *N* subjects, we found that OW/O in children and adolescents was not associated with increased aortic stiffness. Based on our results, we may conclude that the pathophysiological consequences in the circulatory system due to childhood OW/O are partially compensated hemodynamically in these patients, presumably by the opening of small arterioles, by decreasing peripheral arteriolar resistance. Therefore, considering our findings we might speculate that OW/O does not have any direct adverse effect on the arterial function in the by us investigated population. However, it must be emphasized, that in general accumulation of adipose tissue raises the blood pressure—as we also presented in this study—resulting in increased cardiovascular risk in the future. Hence, successful lifestyle intervention programs (focusing on weight reduction, cessation of a sedentary lifestyle, sleeping, stress elimination, and relaxation) may lead to the optimization of the CV risks in the affected subjects. Furthermore, it would be important to follow these OW/O patients by monitoring AFPs, to establish the possible period, when the “haemodynamic compensation/adaptation” of the arterial system might switch into deterioration, resulting in early arterial disease.

The moment, when the compensatory functions in OW/O subjects become insufficient is hardly detectable. For this reason, we aim to design further studies to detect the beginning of this unfavorable vascular function changes.

## Data Availability Statement

The datasets generated for this study are available on request to the corresponding author.

## Ethics Statement

The studies involving human participants were reviewed and approved by Institutional Ethics Committee of the University of Pécs. Written informed consent to participate in this study was provided by the participants' legal guardian/next of kin.

## Author Contributions

All authors listed have made a substantial, direct and intellectual contribution to the work, and approved it for publication.

## Conflict of Interest

MI shares a part in TensioMed Company (Budapest, Hungary), which produces Arteriograph and other medical devices. The remaining authors declare that the research was conducted in the absence of any commercial or financial relationships that could be construed as a potential conflict of interest.
