# Supplementary material for: Childhood Obesity: Does it Have Any Effect on Young Arteries?
Source: Front Pediatr. 2020 Jul 16;8:389. doi: 10.3389/fped.2020.00389 (PMC7378393; doi:10.3389/fped.2020.00389)
Supplement: Supplementary file 2 [file Image_1.pdf]

Supplementary Material

1. Supplementary Figures

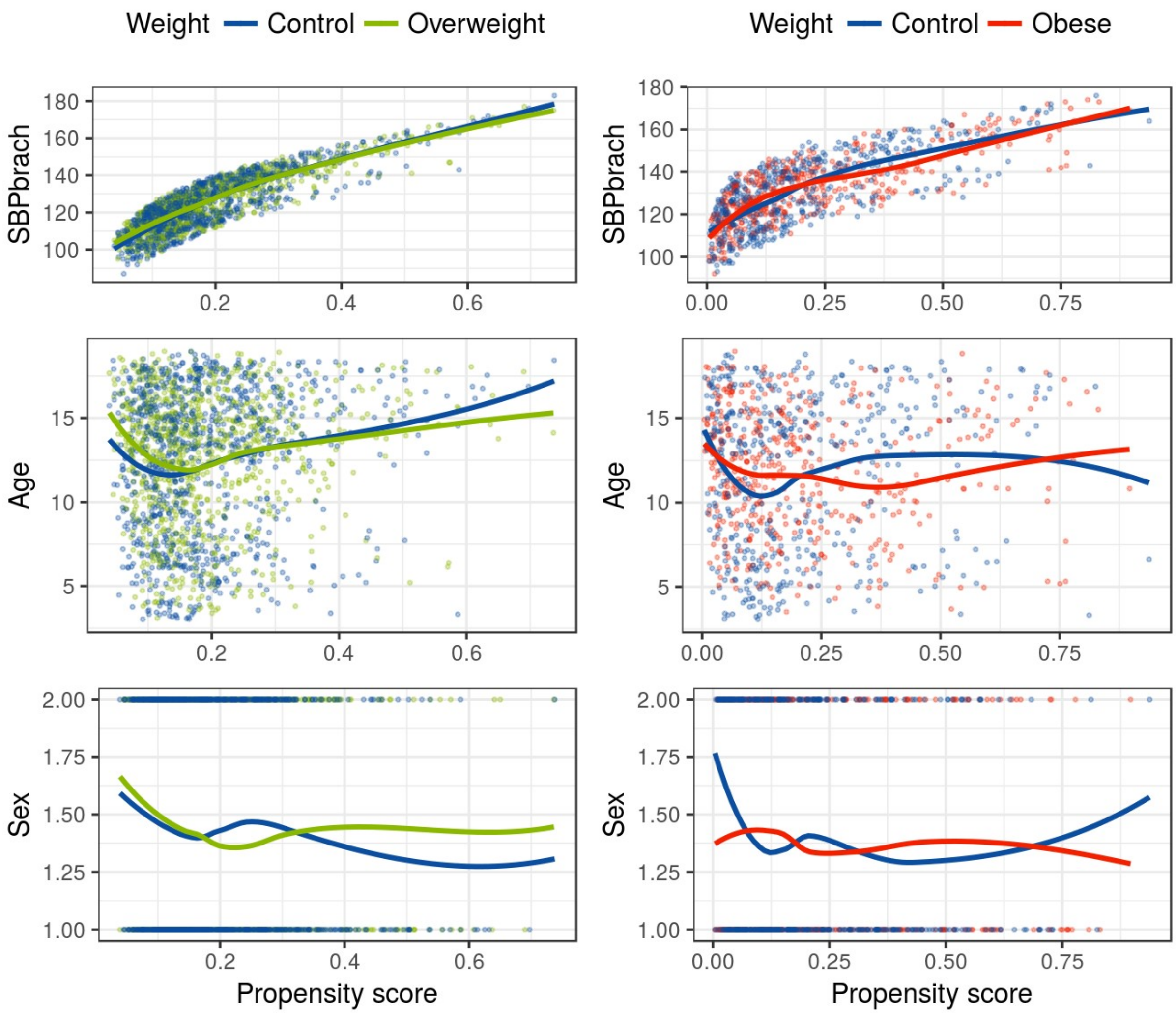

Supplementary Figure 1. Plot of the means of each covariate against the estimated propensity score, separately by weight category
